# Terpenoid Phytosomes as Advanced Delivery Systems: Molecular Interactions, Pharmacological Potential, and Scalable Manufacturing Approaches

**DOI:** 10.3390/ijms27062868

**Published:** 2026-03-22

**Authors:** Shynggys Sergazy, Shyngys Aliakpar, Gulimzhan Adekenova, Khorlan Itzhanova, Orazio Taglialatela-Scafati, Sergazy Adekenov

**Affiliations:** 1 Research and Production Center Phytochemistry, Gazaliyev Street, Karaganda 100009, Kazakhstan; shyngys.aliakpar@nu.edu.kz (S.A.); info@phyto.kz (G.A.); arglabin@phyto.kz (S.A.); 2 School of Pharmacy, Karaganda Medical University, Karaganda 100008, Kazakhstan; itzhanova@kmu.kz; 3 Department of Pharmacy, University degli Studi di Napoli Federico II, 80131 Napoli, Italy; scatagli@unina.it

**Keywords:** terpenoids, phytosomes, phospholipids, molecular complexation, drug delivery systems, natural products

## Abstract

Terpenoids represent a large class of bioactive natural compounds with promising pharmacological properties, including anti-inflammatory, antimicrobial, and anticancer activities. However, their clinical application is often limited by poor aqueous solubility, low membrane permeability, and suboptimal bioavailability. Phytosomal delivery systems have emerged as a promising strategy to enhance the pharmacokinetic performance of plant-derived compounds by forming molecular complexes between bioactive molecules and phospholipids. This review critically examines the structural principles, preparation methods, physicochemical characterization, and biological performance of terpenoid phytosomes. Particular attention is given to the molecular interactions between terpenoids and phospholipids that govern complex formation and vesicular assembly. The review also summarizes current analytical techniques used to confirm phytosome formation and discusses the influence of formulation parameters, including phospholipid composition and molar ratios, on stability and biological activity. In addition, emerging insights from molecular modeling and membrane interaction studies are considered to better understand the mechanisms underlying improved drug delivery. Finally, challenges related to safety assessment, manufacturing scalability, and clinical translation of phytosomal systems are discussed. Overall, terpenoid phytosomes represent a promising nanodelivery platform capable of improving the pharmacokinetic profile and therapeutic potential of terpenoid compounds.

## 1. Introduction

**Figure 1:**
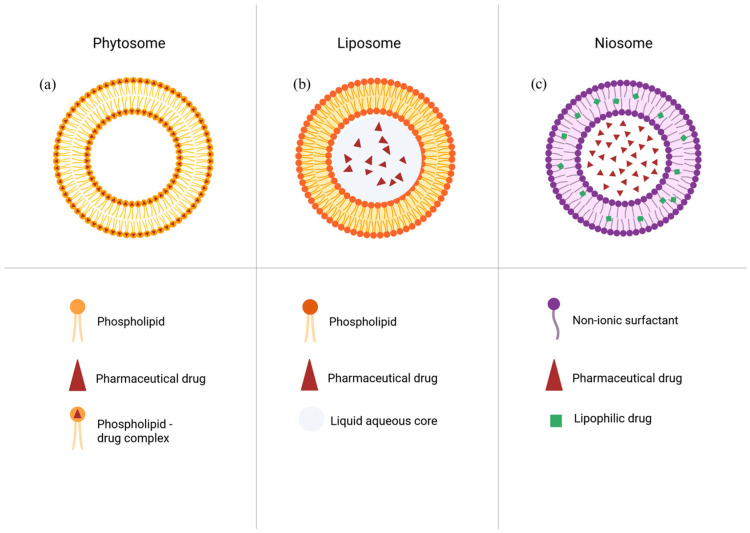
Schematic representation of terpenoid delivery systems: (**a**) phytosomes, displaying the stoichiometric chemical complexation between terpenoids and phospholipid headgroups; (**b**) liposomes, with the drug either in the aqueous core or the lipid bilayer; (**c**) niosomes, where hydrophilic drugs are encapsulated in the aqueous core, while lipophilic terpenoids are entrapped within the hydrophobic intermembrane space of the non-ionic surfactant bilayer.

Terpenoids or isoprenoids represent the largest and most structurally diverse class of natural compounds, with more than 80,000 compounds isolated from plants and algae. From a pharmacological view, terpenoids are attractive drug candidates because they exhibit a spectrum of biological activities, including antioxidant, anti-inflammatory, anticancer, hepatoprotective and wound healing effects [1]. Despite preclinical validation of terpenoids as supplementations, the clinical and commercial translation of terpenoids remains limited. When taken orally, terpenoids are characterized by poor aqueous solubility, strong crystallinity, and chemical and oxidative instability followed by rapid metabolic clearance. To address those challenges, different lipid-based drug delivery systems have been explored, including phytosomes, liposomes and niosomes (Figure 1). While these approaches can improve solubility and stability to a certain degree, they often suffer from drawbacks such as low drug loading, burst release, physical instability, complex manufacturing processes and limited scalability [2].

**Figure 2:**
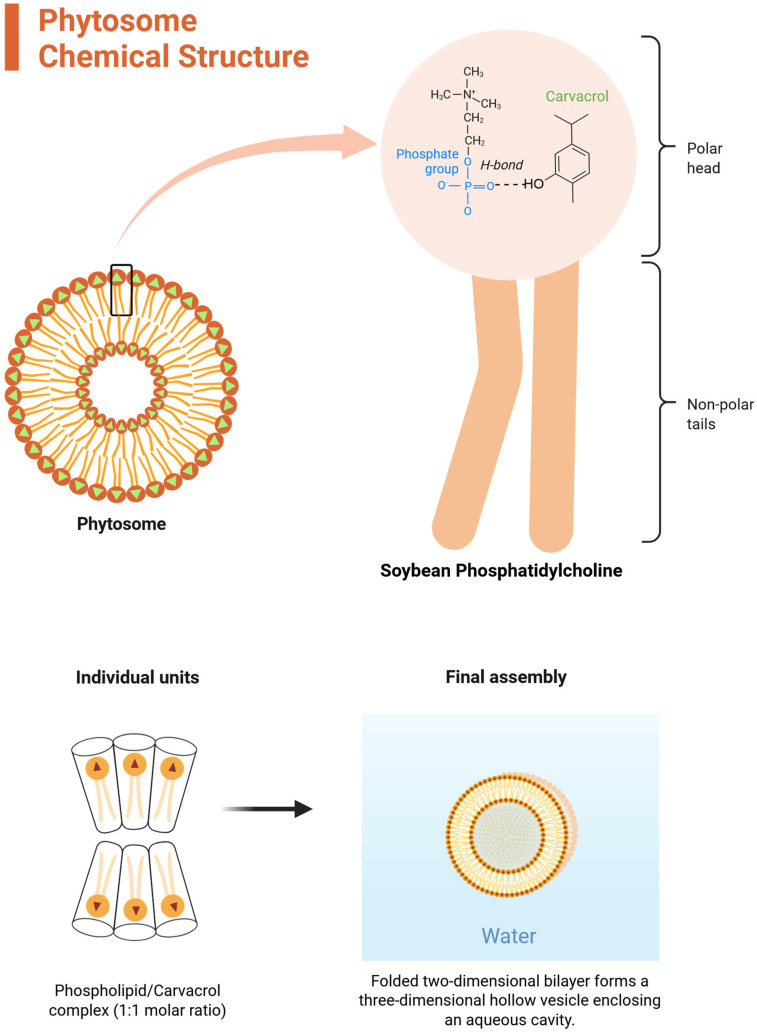
Schematic representation of the self-assembly of terpenoid phytosomes. Carvacrol molecules interact with the phosphate group via hydrogen bonding, consistent with findings reported by [4]. The molar ratio may vary in favor of Phosphatidylcholine to achieve optimal size.

Phytosomes (**a**) have emerged as a novel platform for plant-derived compounds. In this review, a phytosome is defined as a stoichiometric molecular complex formed between a phospholipid (most commonly phosphatidylcholine) and a bioactive compound via non-covalent interactions. Upon dispersion in aqueous media, these amphiphilic complexes can self-assemble into vesicular structures (Figure 2). This mechanistic basis distinguishes phytosomes from conventional lipid carriers such as liposomes, which encapsulate hydrophilic compounds within their aqueous core and incorporate lipophilic or amphiphilic molecules within the phospholipid bilayer. In contrast, phytosomes originate from defined phospholipid–bioactive molecular complexation that precedes vesicle formation [3].

Liposome (**b**) is a well-established technology with FDA-approval precedents [4]. The core principle is to entrap active substances within the inner cavity of liposomes and thus increase the bioavailability of drugs. The materials for liposomes are natural phospholipids: phosphatidylcholine and phosphatidylinositol from soybean and sunflower seeds. The main limitations of liposomal formulation are the stability and pH sensitivity, as well as the amount of substance loaded. In the Research and Production Center, “Phytochemistry” liposomal formulations were created for the Arglabin (**2**) drug. The Arglabin liposomal formulations demonstrated impressive stability profiles; at −10 °C, the formulation remained stable for up to one year. At 20 °C, stability was dependent on the concentration of the drug and reached 90 d for 1.25 mg/mL Arglabin and 5 d for 50 mg/m [5,6]. This stability data indicates significant potential for industrial manufacturing, ensuring both reproducibility and scalability. In addition to Arglabin, products like Doxil have demonstrated the immense therapeutic benefits of lipid encapsulation, serving as a landmark success in the field [7].

Niosomes (**c**) are vesicular delivery systems primarily made of non-ionic surfactants, frequently combined with cholesterol to increase membrane stiffness and minimize cargo leakage. They serve as effective substitutes for liposomes in industrial pharmacy because they provide superior physical and chemical stability, are cheaper to produce, and are less prone to oxidative damage [8]. Since niosomes are not composed of natural polymers, they face significant issues with biocompatibility compared to phytosomes. This is primarily because niosomes are made of synthetic surfactants rather than naturally occurring lipids [9]. The surfactants are commercially available Spans or Tweens, and the specific Hydrophilic–Lipophilic Balance (HLB) of these components dictates the resulting size, layer count, and drug-loading capacity of the niosome. Meantime, cholesterol is vital for adjusting the fluidity and transition temperatures of the membrane to improve overall structural integrity. Furthermore, contemporary studies are investigating blended surfactant formulations and the integration of polymers or charged lipids to refine stability and enable surface modifications for site-specific targeting [10]. Table 1 summarizes the structural difference and rationale behind each of the lipid-based formulations discussed.

In addition to the lipid-based systems described above, several other vesicular nanocarriers have been developed to improve the delivery of poorly soluble bioactive compounds, including transfersomes, ethosomes, and cubosomes. Transfersomes are ultra-deformable vesicles composed of phospholipids and edge activators (typically surfactants) that enhance membrane flexibility and enable efficient transdermal penetration through narrow intercellular spaces of the stratum corneum. Ethosomes are phospholipid vesicles containing high concentrations of ethanol, which increases membrane fluidity and facilitates enhanced skin permeability of encapsulated molecules. Cubosomes, in contrast, represent nanostructured particles derived from bicontinuous lipid cubic phases that form three-dimensional lipid bilayer networks capable of incorporating both hydrophilic and hydrophobic compounds. While these nanocarriers are effective in improving drug delivery, they primarily rely on physical encapsulation mechanisms. In contrast, phytosomes are characterized by the formation of stoichiometric phospholipid–bioactive molecular complexes, typically mediated by hydrogen bonding between functional groups of the phytoconstituent and the polar headgroups of phospholipids, which fundamentally distinguishes them from conventional vesicular delivery systems [11,12,13,14].

Several recent review and research articles have highlighted the growing importance of phytosomal and related nanodelivery systems for improving the bioavailability and therapeutic performance of natural compounds. The recent literature has addressed nanoformulated terpenoids in cancer, phytosome-based complementary therapy for metabolic disorders, phytosomes as a nanotechnology platform for topical delivery of phytochemicals, and preclinical development of phytosomal systems for obesity treatment. However, these works mainly focus on disease-specific applications, broad phytochemical delivery, or individual formulation case studies. In contrast, the present review specifically focuses on terpenoid-based phytosomes and emphasizes the structural principles of terpenoid–phospholipid complex formation, the physicochemical and analytical methods used to confirm phytosome formation, and the formulation parameters influencing pharmacokinetic enhancement and translational potential. By integrating structural chemistry, characterization strategies, pharmacological evidence, and manufacturing considerations, this review aims to provide a more mechanistic and formulation-orientated perspective on terpenoid phytosomes than the existing recent literature [15,16,17].

Despite growing interest in phytosomal delivery systems, the mechanisms governing terpenoid–phospholipid interactions, their physicochemical behavior, and their implications for pharmacokinetics remain incompletely systematized. Therefore, the aim of this review is to critically analyze the structural basis of terpenoid–phospholipid complex formation, summarize current strategies for the preparation and characterization of terpenoid phytosomes, and evaluate their pharmacological potential and translational prospects. Particular emphasis is placed on mechanistic aspects of molecular interactions, physicochemical determinants of bioavailability enhancement, and emerging approaches including molecular modeling for understanding phytosome–membrane interactions.

**Table 1:** Lipid-based drug delivery systems.

System Type	Structure	Suitability for Terpenoids	Reference
Phytosome	The terpenoid is associated with the phospholipid headgroup through non-covalent interactions, primarily hydrogen bonding.	Great for polar terpenoids (e.g., boswellic acids).	[18]
Liposome	The terpenoid is physically trapped inside the lipid bilayer or the water core.	Bypassing presystemic metabolism.	[19]
Niosome	Similar to a liposome but made of non-ionic surfactants instead of phospholipids.	Increasing stability and reducing costs.	[20]

## 2. Terpenoids as Bioactive Molecules: Structural and Physicochemical Determinants Relevant to Phytosome Formation

Terpenoids are structurally diverse secondary metabolites, biologically synthesized from isoprene units via the mevalonate (MVA) and methylerythritol phosphate (MEP) pathways by plants and algae. Their classification into mono-(C10), sesqui-(C15), di-(C20), tri-(C30), tetra-(C40), and polyterpenoids reflects carbon skeleton length and also differences in molecular rigidity, polarity, and functional group density, which collectively govern pharmacological behavior and formulation feasibility [21].

From a pharmacokinetic standpoint, terpenoids exhibit unfavorable physicochemical properties such as high lipophilicity (commonly logP > 3–5), low aqueous solubility, strong crystal lattice energy, and limited ionizability, which together result in dissolution rate-limited absorption in the human body [22]. Additionally, their distribution is complicated by chemical instability, specifically susceptibility to oxidation, photodegradation and extensive first-pass metabolism mediated by cytochrome P450 enzymes [23]. Well-studied examples such as boswellic acids, andrographolide, ursolic acid and carvacrol consistently demonstrate poor and highly variable systemic exposure following oral administration.

Importantly, not all terpenoids are equally problematic from a formulation perspective. A critical but often underappreciated determinant of delivery system compatibility is the presence, type, and spatial orientation of polar functional groups embedded within otherwise hydrophobic terpene scaffolds. Hydroxyl, carboxyl, carbonyl, epoxide, and lactone functionalities introduce hydrogen bond donor and acceptor capacity, which profoundly influences intermolecular interactions with excipients [24].

Terpenoids lacking such functionalities behave as purely lipophilic oils and are poorly suited for complexation-based delivery systems. Conversely, terpenoids containing strategically positioned polar groups such as the hydroxyl groups in certain triterpenes exhibit enhanced affinity for amphiphilic carriers like phospholipids [24,25,26]. This distinction explains why certain terpenoids including boswellic acids and andrographolide derivatives are repeatedly reported as successful candidates for phytosome formulation, while others remain confined to simpler emulsified or encapsulated systems [27,28].

In addition to functional group chemistry, molecular flexibility and conformational adaptability play decisive roles in phytosome compatibility. Rigid, planar molecules with limited torsional freedom may form fewer stable complexes due to steric constraints, whereas moderately flexible terpenoids can align more effectively with phospholipid headgroups, maximizing hydrogen bonding and van der Waals interactions [24]. This structural adaptability contributes to reduced crystallinity, enhanced amorphization, and improved miscibility within lipid environments, which collectively underpin the superior dissolution and absorption profiles observed for phytosome-based systems compared to free terpenoids or simple physical mixtures [29].

Physicochemical and structural characteristics of terpenoids define both the necessity for advanced delivery systems and the rational basis for selecting phytosome technology. Rather than serving as a universal solution for all terpenoids, phytosomes represent a targeted strategy for a chemically defined subset of terpenoids whose molecular features enable stable phospholipid complexation and meaningful pharmacological enhancement.

## 3. Current Advancements in Terpenoid Phytosomes for Enhanced Delivery

**Figure 3:**
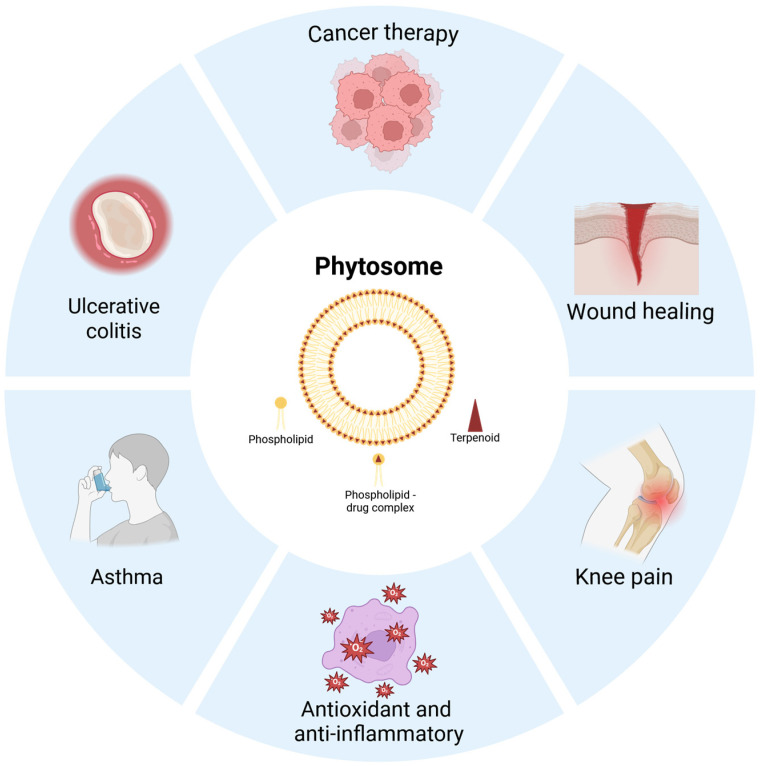
Applications of terpenoid phytosomes.

The chemical structures of the terpenoid formulated as phytosomes and their pharmacological activities are summarized in Table 2 and Table 3. Application of terpenoid phytosomes are illustrated in Figure 3.

### 3.1. Diterpenoid Lactones

Andrographolide (AG) (**1**) is a diterpenoid lactone with recognized anti-inflammatory and anticancer activity, but its clinical utility is limited by poor solubility and restricted cellular permeability. To improve its delivery, AG-loaded phytosomal nanovesicles (AG-PTMs) were developed and optimized using phosphatidylcholine, yielding an optimized formulation with a particle size of 243.7 ± 7.3 nm, a polydispersity index of 0.310, and an entrapment efficiency of 72.20 ± 4.53%. In HepG2 liver cancer cells, AG-PTMs demonstrated significantly enhanced cellular uptake compared with free AG, reaching 35.30 ± 2.20% and 74.00 ± 5.10% at 2 and 4 h, respectively, whereas AG-Raw showed uptake values of 15.20 ± 1.10% and 32.10 ± 2.50% at the same time points. This increase in intracellular delivery was associated with markedly improved antiproliferative activity, as reflected by a reduction in IC50 from 14.09 ± 1.81 µM for free AG to 4.02 ± 0.14 µM for AG-PTMs, together with enhanced G2/M arrest, increased pre-G1 apoptotic fraction, higher Annexin V-positive cell populations, increased ROS generation, mitochondrial membrane depolarization, upregulation of BAX and caspase-3, and downregulation of BCL2. Importantly, however, the study did not directly investigate the specific uptake routes responsible for AG-PTM internalization. Therefore, although the superior cellular accumulation of AG-PTMs is consistent with energy-dependent endocytic uptake commonly described for nanosized lipid-based systems, no direct evidence was provided to distinguish whether clathrin-mediated, caveolae-mediated, or other endocytic pathways were preferentially involved compared with free andrographolide [30].

**Table 2:** Natural Terpenoids.

No.	Compound Name	Class	Physical and Chemical Constants	Functional Group	Structural Formula	Reference
**1**	Andrographolide	Diterpenoid lactone	C_20_H_30_O_5_ m.p. 230–231 °C [α]_D_ − 126°	Lactone ring, hydroxyl groups	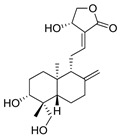	[31,32]
**2**	Arglabin	Sesquiterpene lactone	C_15_H_18_O_3_ m.p. 100–102 °C [α]_D_ + 45.6°	Exocyclic methylene, lactone carbonyl	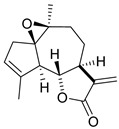	[33]
**3**	α-boswellic acid	Triterpenoid	C_30_H_48_O_3_ m.p. 268–270 °C [α]_D_ + 80°	Carboxyl, hydroxyl groups	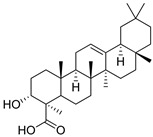	[34,35]
**4**	β-boswellic acid	Triterpenoid	C_30_H_48_O_3_ m.p. 234–236 °C [α]_D_ + 105°	Carboxyl, hydroxyl groups	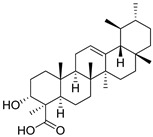	[34,35,36]
**5**	11-keto-β-boswellic acid	Triterpenoid	C_30_H_46_O_4_ m.p. 196–198 °C [α]_D_ + 78.5°	Carboxyl, hydroxyl groups	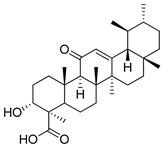	[34,37,38]
**6**	Carvacrol	Monoterpenoid	C_10_H_14_O m.p. 1 °C b.p. 237.7 °C	Phenolic OH	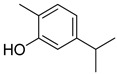	[39,40]
**7**	Ursolic acid	Triterpenoid	C_30_H_48_O_3_ m.p. 284 °C [α]_D_ +67.5°	Carboxyl, hydroxyl groups	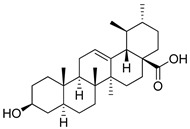	[41,42]

### 3.2. Pentacyclic Triterpenoids

#### 3.2.1. Boswellic Acids

Boswellic acids (BAs) (**2**,**3**,**4**) are constituents of Boswellia serrata gum resin with potent anti-inflammatory properties, primarily mediated through non-redox inhibition of 5-lipoxygenase (5-LOX) and suppression of pro-inflammatory mediators such as TNF-α and IL-1. Therapeutically, BAs have shown activity in chronic inflammatory conditions including osteoarthritis, rheumatoid arthritis, asthma, and inflammatory bowel disease [43,44]. However, the clinical applicability of conventional Boswellia extracts is limited by poor aqueous solubility, low intestinal permeability, and consequently low systemic exposure of key bioactive constituents such as 11-keto-β-boswellic acid (KBA) and 3-acetyl-11-keto-β-boswellic acid (AKBA) [18,45].

To address these limitations, a lecithin-based boswellic acid phytosomal complex, commercially available as Casperome^®^, was developed. In this formulation, boswellic acids form a phospholipid complex with lecithin/phosphatidylcholine, which is thought to improve their dispersion in gastrointestinal fluids and facilitate transport across biological membranes [18]. Comparative pharmacokinetic studies showed that this phytosomal formulation increased systemic exposure, with plasma levels of KBA and AKBA reported to be up to seven-fold and three-fold higher, respectively, than those achieved with the unformulated extract [16,18]. In addition, tissue distribution studies suggested greater delivery of boswellic acids to otherwise difficult-to-reach organs, including the brain and lungs [18,46].

From a pharmaceutical perspective, phospholipid complexation may also contribute to improved stability of boswellic acids by enhancing dispersion and protecting bioactive constituents from the aggressive gastrointestinal environment, an effect that has been described more generally for phytosomal systems [18]. However, direct published data on the long-term storage stability of this specific boswellic acid phytosomal complex, or on its behavior under different gastrointestinal pH conditions, remain limited in the literature. Accordingly, although the available evidence supports improved absorption and likely improved formulation robustness, these aspects still require more explicit pharmaceutical characterization.

Clinical studies nevertheless support the therapeutic relevance of boswellic acid phytosomal delivery. In patients with irritable bowel syndrome, the phytosomal formulation reduced abdominal pain, bloating, and localized inflammation compared with standard management [47]. In ulcerative colitis in remission, it was associated with reduced intestinal permeability and improvement in occult blood parameters [44]. In musculoskeletal settings, boswellic acid phytosomal supplementation has been reported to reduce edema and support functional recovery in tendon and sports-related conditions and to reduce postoperative edema and seroma when combined with other nutraceuticals [43,48]. Beneficial effects have also been described in temporomandibular disorders, where supplementation was associated with reduced pain and improved joint mobility [49]. In a registry study of 52 young rugby players, boswellic acid supplementation improved physical function, increased pain-free walking distance, and reduced inflammatory (CRP) and cartilage damage (COMP) markers, while lowering the need for rescue NSAIDs [50]. Taken together, these findings indicate that phytosomal complexation substantially improves the pharmacokinetic and clinical applicability of boswellic acids, while also highlighting the need for more detailed stability and formulation studies.

#### 3.2.2. Ursolic Acid

Another pentacyclic terpenoid, ursolic acid (UA) (**7**), was successfully incorporated into polymer-functionalized phytosomal vesicles (UA-PLL-HA.P), which were further modified with poly-L-lysine (PLL) and hyaluronic acid (HA) to create a targeted delivery system responsive to acidic pH and capable of receptor-mediated interaction with CD44-expressing cells. Cellular uptake was significantly higher in CD44-positive SCC-7 cells than in CD44-negative BT-474 cells, which was attributed to the active targeting effect of hyaluronic acid toward the CD44 receptor. In cytotoxicity assays, the modified phytosomal vesicles showed the strongest antiproliferative activity. At a concentration of 100 μg/mL, the survival rate of SCC-7 cells was approximately 33%, compared with about 55% for free UA. In vivo studies in Balb/c nude mice further demonstrated significant inhibition of tumor growth, indicating that the modified phytosomal system effectively delivered ursolic acid into tumor tissue. Moreover, no obvious toxicity or adverse effects were observed in the treated animals [51].

In a separate study, ursolic acid phytosomes were evaluated for hepatoprotective activity and bioavailability in Wistar rat models with CCl4-induced liver injury [52]. The animals received either free ursolic acid extract or the phytosomal formulation at doses of 10 and 20 mg/kg (orally), equivalent to pure ursolic acid, for 7 days. To induce liver damage, researchers made a single intraperitoneal injection of a CCl4 and olive oil mixture. They found that the phytosomal form increased the serum bioavailability of ursolic acid by more than 8-fold and extended its elimination half-life 12-fold compared to the pure compound at the same dose. Interestingly phytosomes showed slightly better hepatoprotective effect measured by decrease in serum AST, ALT and ALP activity and serum total bilirubin levels significantly decreased only in the group receiving phytosomes. The activity of hepatic antioxidant enzymes (SOD, CAT, GPx, GST, and GR), GSH levels, and total liver protein concentration were most effectively stimulated by the administration of 20 mg/kg of ursolic acid in phytosomal form. The histological examination of hematoxylin–eosin-stained sections confirmed a significant improvement in liver tissue architecture in the phytosomal group compared to both the pure acid and the CCl4 control groups. These preclinical data provide an argument for the use of phytosomal forms of bioactive compounds in the treatment of liver diseases.

### 3.3. Monoterpenoid

Carvacrol (CAR) (**6**) faces issues of high volatility and rapid metabolic clearance. In this instance, carvacrol-loaded phytosomes (CLNPs) have been engineered as advanced vesicular delivery systems. Optimized CLNPs formulated with Phospholipon 90H achieved a high encapsulation efficiency of 92.35% and showed a sustained release profile compared to free carvacrol [53]. Combined with hydrogel, CLNPs were adapted for topical administration in wound healing. Hydrogel significantly accelerated wound healing in vivo by promoting cellular proliferation, increasing collagen deposition, and upregulating growth factors like TGF-β1 and VEGF [53]. Histological finding suggested that the CAR-loaded phytosomes could induce cell proliferation and support cellular regeneration by PCNA modulation, which means CAR has tissue remodeling and wound healing capacity. In the field of sperm cryoconservation, CLNPs have proven highly effective in enhancing the cryotolerance of buffalo semen. Supplementing sperms with CLNPs at a concentration of 5 µg/mL significantly reduced oxidative stress markers, such as malondialdehyde (MDA), while maintaining mitochondrial membrane potential and improving post-thaw sperm motility and pregnancy rates (61.5% vs. 38.5% in control) [54]. By stabilizing the phenolic terpene within a stoichiometric phospholipid complex, phytosomes ensure protected biological activity and targeted delivery, supporting their potential as an effective platform for the administration of volatile phytochemicals.

**Table 3:** Representative Terpenoid Phytosomes: Structural Features and Applications.

Phytosome	Composition	Preparation Method	Particle Size/PDI/Zeta	Pharmacological Activities	Reference
Andrographolide (AG) phytosomal nanovesicles (AG-PTMs)	AG:Soybean l-α-phosphatidylcholine (95%) 1:2.7	Thin-film hydration	243.70 ± 7.30 nm, 0.31, no data on zeta potential	Anti-inflammatory, antiviral	[55]
Boswellic acids (BA)-loaded Casperome^®^	BA:Soy lecithin 1:1	Proprietary	Proprietary	Anti-inflammatory on the colon, anti-arthritic, antidiarrheal activity	[56,57]
Carvacrol (CAR)-loaded phytosomes (CLNPs)	CAR: LIPOID S100 (soybean phosphatidylcholine) 1:2	Thin-film hydration	110.1 ± 20.8, 0.23 ± 0.04, −15.9 ± 3.99 mV	Wound healing, antioxidant activity,	[53]
Ursolic acid (UA) polymer-functionalized phytosomal vesicles (UA-PLL-HA. P)	Cholesterol (Chl): phosphatidylcholine (PC): UA 2:1:0.5 + poly L and hyaluronic acid (HA)	Ethanol injection method followed by centrifugation and sonification	102.0 ± 3.0 nm, 0.254 ± 0.028, −8.5 ± 1.1 mV	Stimulus-responsive antitumor activity	[16,51]

## 4. Physicochemical Characterization of Terpenoid Phytosomes

The characterization of terpenoid phytosomes is essential for confirming the formation of phospholipid–bioactive molecular complexes and for distinguishing these systems from simple physical mixtures or conventional lipid vesicles. Because phytosomes originate from stoichiometric interactions between bioactive compounds and phospholipid headgroups, multiple complementary analytical techniques are typically employed to verify molecular complexation, structural modification, and vesicle formation.

Spectroscopic techniques provide direct evidence of molecular interactions between terpenoids and phospholipids. Fourier-transform infrared spectroscopy (FTIR) is frequently used to identify hydrogen bonding or other intermolecular interactions between functional groups of the bioactive compound and phospholipid headgroups. For example, in the development of carvacrol-loaded phytosomes, shifts in hydroxyl stretching and aromatic vibration bands were observed when compared with the spectra of the free compound and phosphatidylcholine, supporting the formation of a stable phospholipid–terpenoid complex [53]. Similar spectral changes have been reported in other phytosomal formulations, where modifications in band intensity or position reflect interactions between bioactive molecules and phospholipid headgroups [58,59]

Nuclear magnetic resonance spectroscopy provides additional insight into molecular organization within phytosomal systems. Changes in proton or carbon chemical shifts may occur when bioactive molecules interact with phospholipid headgroups or become incorporated into lipid environments. Studies investigating phenolic terpenes interacting with phospholipid bilayers have demonstrated that such compounds can modify membrane structure and intermolecular interactions within the lipid matrix [60]. These observations support the concept that phytosomes represent structured phospholipid–bioactive complexes rather than simple dispersions of phytochemicals.

Thermal analysis techniques also provide important evidence of phytosome formation. Differential scanning calorimetry (DSC) allows the detection of thermodynamic changes associated with interactions between bioactive compounds and phospholipid membranes. In many phytosomal systems, the characteristic melting peak of the crystalline phytochemical disappears or becomes broadened following complexation with phosphatidylcholine, indicating reduced crystallinity and molecular dispersion within the lipid phase. Similar thermal behavior has been observed in lipid-based systems containing triterpenoid compounds, where interactions with the phospholipid matrix modify membrane organization and thermal transitions [61].

In addition to confirming molecular complexation, physicochemical characterization of the resulting vesicles is necessary to evaluate dispersion stability and formulation performance. Particle size and size distribution are commonly determined by dynamic light scattering (DLS), which measures fluctuations in scattered light caused by Brownian motion of nanoparticles in suspension [62]. Phytosomal formulations typically exhibit nanoscale particle sizes ranging from approximately 80 to 300 nm with relatively low polydispersity index values, indicating homogeneous vesicle populations. Surface charge is commonly evaluated through zeta potential measurements, which provide information about electrostatic stabilization and colloidal behavior of nanoparticles [63].

One of the critical parameters influencing phytosome formation is the molar ratio between the terpenoid compound and the phospholipid component. This ratio determines the extent of intermolecular interactions between the bioactive molecule and the phospholipid headgroups and therefore strongly influences the stability and physicochemical properties of the resulting vesicles. Most phytosomal formulations reported in the literature employ terpenoid-to-phosphatidylcholine molar ratios ranging from approximately 1:1 to 1:2, which appear to provide sufficient phospholipid content to stabilize the molecular complex while maintaining efficient loading of the bioactive compound [58]. Deviations from these ratios may significantly influence the formation of hydrogen bonding interactions between the terpenoid functional groups and phospholipid phosphate moieties, thereby affecting thermodynamic stability, particle size distribution, and vesicle morphology. Experimental studies have demonstrated that increasing phospholipid content can improve vesicle stability and reduce particle size, whereas insufficient phospholipid concentrations may lead to incomplete complexation and reduced formulation stability [53]. Consequently, optimization of the terpenoid-to-phospholipid molar ratio represents a crucial step in phytosome formulation development, as this parameter directly affects complex formation, drug loading efficiency, and the pharmacokinetic behavior of the resulting nanocarrier systems.

Finally, stability assessment represents an important component of phytosome characterization. Accelerated stability studies conducted under varying environmental conditions are often used to evaluate potential changes in particle size, surface charge, and drug loading capacity during storage, providing insight into the robustness of the phospholipid–bioactive complex and the long-term stability of the formulation [64].

Representative physicochemical characteristics reported for terpenoid phytosomal formulations, including particle size distribution, polydispersity index, and zeta potential values, are summarized in Table 4.

## 5. Manufacturing Technologies for Terpenoid Phytosomes: Process Design, Scalability, and Regulatory Implications

Despite various applications of phytosomes, the manufacturing of terpenoid phytosomes is a translational challenge. Scaling laboratory methods to full-scale production needs to be optimized to ensure they are reproducible, scalable, and compliant with pharmaceutical quality standards such GMP. For example, the manufacturing method has to achieve a polydispersity index lower than 0.3 to ensure uniformity of phytosome size and stability profiles. Phospholipid properties and molar percent are important as well, as they determine the geometry and fluidity of phytosome [53].

Nowadays, there are three major methods used for phytosome formation between phospholipids and terpenoids: thin-layer hydration, co-solvency and salting out (Table 5).

### 5.1. Thin-Film Hydration

**Figure 4:**
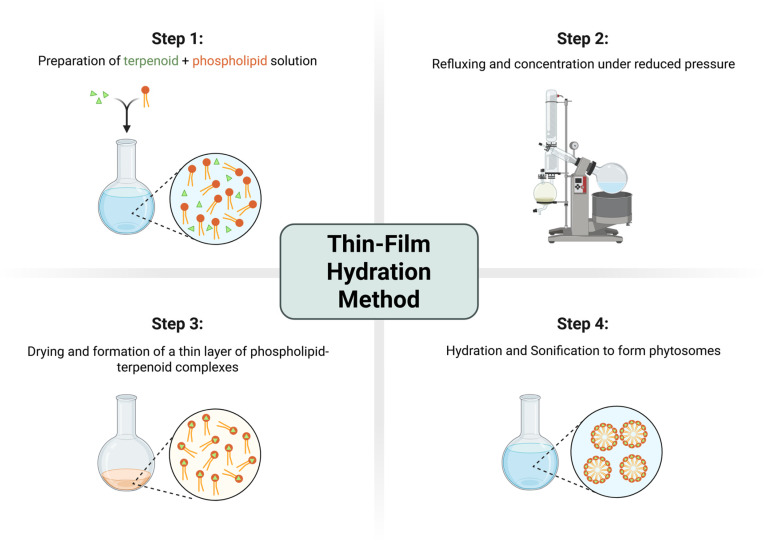
Steps of thin-layer hydration method.

Thin-film formation is the most widely reported method for phytosome preparation in academic studies [30,45,53,58]. Typically, the terpenoid and phosphatidylcholine are dissolved in aprotic or protic organic solvents such as ethanol, methanol, acetone, or dichloromethane and then stirred magnetically overnight to allow the complex to begin forming followed by solvent removal in Rotavapor to make a dry film. This dry film is stored overnight and then hydrated by adding water and using sound waves to break the film into phytosome. Among other methods, thin-layer preparation gives the smallest particle size and provides a larger surface layer, as well as a convenient time for phytosomal layer formation. Small particles are less prone to aggregate and more stable for storage. Rotational movement of the flask prevents aggregation, as well, and increases the thickness and homogeneity of the formed film. An additional thing to consider is that phytosomes obtained through thin-layer hydration depict highest zeta potential, which indicates stronger repulsion and reduces chances of sudden aggregation. Researchers achieved a polydispersity index of 0.1 to 0.25 at 66.7 molar percent of the lipid and low saturation, which indicates uniform phytosome formation. The tin-layer method is simple, flexible in terms of solvent choice, and does not require relatively high-end equipment [69]. However, thin-layer formation is highly sensitive to variations in temperature, evaporation rate, solvent polarity, and component ratios and saturation of the lipid, all of which influence the kinetics of complex formation, polydispersity and particle size. Thin-film hydration method is illustrated in Figure 4.

**Table 5:** Manufacturing Technologies for Terpenoid Phytosomes: Process Characteristics, Scalability, and Translational Relevance.

Manufacturing Approach	Core Process Principle	Advantages	Limitations	Scalability Perspective	References
Thin-film (rotary evaporation)	Co-dissolution of terpenoid and phospholipid followed by controlled solvent removal	The process provides a larger surface area and sufficient time for layer formation, preventing particles from clumping	The process is long, requiring overnight magnetic stirring and another overnight refrigeration step	Highly scalable, yet time-intensive and equipment-demanding	[30,45,58,70]
Co-solvency	Terpenoids are dissolved in a blend of water-miscible solvents	It is less complex than thin-film hydration and avoids the need for specialized equipment like a rotary evaporator to form a dry film	It generally results in larger particle sizes than the thin-film hydration method	Highly scalable due to simplicity	[53,58,71]
Salting out	Rapid precipitation of phytosome complex in a non-solvent phase	Good particle size control and high saturation and molar percent of lipids	Largest particles and least uniform at low lipid levels	Moderate. Purity can be compromised due to residual salt presents	[53,72,73]

### 5.2. Co-Solvency

The second major method is co-solvency; it technically is even more simple than the thin-film layer method and relies on gradual mixing of both solutions and passive evaporation [71]. In separate beakers, terpenoid and phospholipid are dissolved in ethanol. Then, a phospholipid solution is added to the terpenoid while on a magnetic stirrer. Finally, the mixture solvent is evaporated and hydrated with distilled water followed by sonification. Despite this method being less time-consuming, co-solvency produces bigger particles and is far more dependent on stoichiometry of phosphatidylcholine and terpenoid, as well as the saturation of lipids to maintain the uniformity of phytosomes and their homogeneity. Lower zeta potential should also be considered as a reason for aggregation in co-solvency.

### 5.3. Salting Out

The third method is salting out, which uses n-hexane to precipitate and separate phytosomes after dissolution and stirring. Interestingly, it increases the molar percent of phospholipid and its saturating has the opposite effect of PDI compared to thin-layer and co-solvency. The salting out method is most demanding in terms of the amount and saturation of the lipids since it generally produces big and relatively unstable particles [53]. Another comparative study demonstrated salting out as a disadvantaged method with the largest particle size (within the range of 281.86 nm and 466.06 nm) compared to co-solvency (63.10 nm to 369.56 nm) [74].

Despite growing interest in phytosomal technologies, clearly documented examples of terpenoid phytosomes that have progressed from laboratory protocols to fully described GMP-compliant pharmaceutical manufacturing remain scarce in the published literature. Apart from lecithin-based boswellic acid products, most reported systems remain at the experimental or preclinical stage, and quantitative data on industrial throughput, production cost, process validation, and regional regulatory classification are rarely disclosed. In practice, this lack of transparency makes it difficult to compare true translational readiness across formulations. Future progress in this field will therefore depend not only on formulation optimization but also on better reporting of scale-up parameters, process economics, GMP adaptation, and product classification pathways across different regulatory jurisdictions.

## 6. Discussion

Various studies demonstrate that phytosomes loaded with boswellic acids, ursolic acid, carvacrol, and andrographolide represent successful examples of vesicular delivery systems that are able to reach clinical trial (Table 6). Their enhanced pharmacological performance stems from hydrogen bonding between the polar functional groups of these terpenoids and phospholipid headgroups. Consequently, phytosome technology should be viewed as a structure-guided strategy: it is most effective for terpenoids possessing the necessary polar chemical groups to form complexes, effectively transforming natural substances from poorly soluble crystals or volatile oils into bioavailable, lipid-compatible dispersions [3,18]. This distinction has profound implications for absorption, as phytosomes exhibit enhanced membrane affinity and can exploit membrane-mediated uptake pathways rather than relying exclusively on aqueous dissolution. Evidence from permeability, pharmacokinetic, and tissue distribution studies consistently supports this interpretation, with phytosome formulations achieving higher plasma exposure and prolonged systemic residence compared to free terpenoids [18]. Although available studies consistently suggest improved efficacy of phytosomal formulations, cross-study comparison remains difficult because safety endpoints, dose–response relationships, and therapeutic windows have not yet been reported in a sufficiently standardized manner.

Another critical insight emerging from the literature is that the pharmacological amplification observed with terpenoid phytosomes is not merely quantitative but often qualitative. In multiple therapeutic models including inflammation and wound healing, phytosome formulations elicit biological responses that exceed those predicted from increased bioavailability alone. For example, enhanced induction, pronounced suppression of pro-inflammatory cytokines, and accelerated tissue regeneration have been reported at doses where free terpenoids exhibit marginal or inconsistent effects [53]. This suggests that phytosome-mediated delivery may alter intracellular distribution, drug–target interactions, or signaling pathway regulation [69].

Modern mechanistic approaches such as molecular docking, molecular dynamics simulations, and membrane biophysics can provide additional insight into how terpenoid–phospholipid complexes interact with biological membranes at the molecular level. These methods can be used to predict hydrogen bonding, membrane insertion depth, bilayer perturbation, and the energetic stability of phospholipid–terpenoid assemblies. Among the systems discussed in this review, the most explicit example is carvacrol, for which docking and biophysical analyses suggest favorable interaction with phosphatidylcholine headgroups and membrane regions, supporting the concept that phytosomal complexation may alter membrane affinity and intracellular delivery behavior [53,60]. However, for many terpenoid phytosomes, including andrographolide and boswellic acid systems, direct membrane–protein interaction studies and high-resolution molecular modeling data remain limited. Consequently, although molecular modeling is a promising tool for mechanistic understanding and rational design, this area remains insufficiently developed and deserves greater emphasis in future phytosome research.

The next interesting aspect of the terpenoid phytosomes is the intrinsic biological activity of phosphatidylcholine that may complicate the interpretation of pharmacological outcomes. As a biological membrane component with known hepatoprotective, membrane-stabilizing, and anti-inflammatory properties, phosphatidylcholine may act synergistically with terpenoids, particularly in models of liver disease, metabolic dysfunction, and chronic inflammation. While this synergy may contribute to enhanced therapeutic outcomes, it also introduces an important confounding variable that is rarely controlled for in experimental designs. Many studies compare terpenoid phytosomes exclusively with free terpenoids, without including phospholipid-only controls, thereby limiting mechanistic attribution. Addressing this gap will be essential for showing delivery-related effects of excipient-mediated biological activity [4,76]. Researchers should also perform a head-to-head comparison with other vesicular delivery systems such as liposomes (b) and niosomes (c), because there are unresolved questions regarding relative and real advantages [77,78].

Despite substantial progress, critical knowledge gaps persist that currently limit the broader acceptance of terpenoid phytosomes in mainstream pharmaceutical development. First, the majority of available data are derived from in vitro studies or animal experiments, with a clear lack of clinical trials where phytosomes are not used as supplementation. Second, formulation protocols vary widely across studies in terms of phospholipid type, molar ratios, solvents, and processing conditions, complicating cross-study comparisons and meta-analyses if these protocols are even published. Third, head-to-head comparisons between phytosomes and alternative advanced delivery systems such as liposomes, niosomes or any other carriers are non-existent, leaving unresolved questions regarding relative and real advantages [77,78].

Our understanding of phytosome formation also remains incomplete. Although molecular complexation between terpenoids and phospholipids is supported by spectroscopic and solid-state analyses, the cellular and subcellular fate of phytosome complexes has not been systematically elucidated. Most studies infer improved absorption and efficacy indirectly through pharmacokinetic or pharmacodynamic outcomes rather than directly examining membrane interactions, intracellular trafficking, or target engagement. Moreover, the intrinsic biological activity of phosphatidylcholine—including its membrane-stabilizing, hepatoprotective, and anti-inflammatory properties—represents a potential confounding factor that is rarely controlled for experimentally, as phospholipid-only comparator groups are frequently absent [79].

Safety considerations also deserve greater attention in the context of terpenoid phytosomes. Although phosphatidylcholine and related phospholipids are generally regarded as biocompatible and are widely used in pharmaceutical and nutraceutical formulations, phytosomal complexation may substantially modify exposure profiles, tissue distribution, and local membrane interactions in the incorporated terpenoid. As a result, improved delivery does not necessarily imply an unchanged safety profile. While several studies cited in this review reported no obvious acute toxicity or adverse effects in preclinical models, systematic evaluation of repeated-dose exposure, prolonged administration, organ-specific accumulation, immunological effects, and therapeutic windows remains limited. Future studies should therefore include more rigorous toxicological assessment alongside efficacy testing in order to define the long-term safety boundaries of terpenoid phytosomal systems.

From a technological and translational perspective, manufacturing considerations emerge as a decisive factor in determining the real-world applicability of terpenoid phytosomes. Thin-layer, salting out and co-solvency remain widely used method for laboratory scale production, but these approaches are greatly dependent on the composition of phospholipid used for the phytosome formation and require quality control in each batch to be sure of uniform size and surface charge. Systematic comparisons of these manufacturing routes for each individual terpenoid remain scarce [30,45,53,58].

Regulatory considerations further underscore the need for greater standardization and transparency. While phospholipids are generally recognized as safe and widely used in pharmaceutical and food products, the classification of phytosome formulations varies across jurisdictions, oscillating between dietary supplements, nutraceuticals, and drug products. Clear regulatory pathways will require robust characterization, reproducibility, and clinical evidence demonstrating real benefits over existing formulations. Without such data, considering its risks, phytosome technology remains confined to niche applications despite its compelling rationale [54,57].

Taken together, the literature supports a view of terpenoid phytosomes as a rational, structure-driven formulation with visible advantages. Their successful application depends on careful matching of terpenoid structure to phospholipid chemistry, the appropriate selection of manufacturing technologies, and rigorous biopharmaceutical and pharmacological evaluation. Future advances will likely arise from interdisciplinary efforts that integrate molecular modeling, advanced analytical characterization, scalable manufacturing, and clinical validation, thereby transforming phytosome technology from a promising formulation concept into a mature translational platform.

## 7. Conclusions

The terpenoid phytosomal delivery system is a rational solution to long-standing pharmacokinetic limitations. By addressing the volatility of carvacrol, the poor solubility of andrographolide and ursolic acid, and the low bioavailability of boswellic acids, phytosomes unlock the therapeutic potential of these natural compounds, offering a novel platform for future clinical and industrial translation. At the same time, the field faces important challenges related to standardization, clinical validation, and regulatory alignment. Solutions for these problems will require interdisciplinary collaboration integrating molecular chemistry, pharmaceutical technology and pharmacology. With continued progress in mechanistic understanding, standardization, and clinical validation, terpenoid phytosomes may evolve from promising formulation concepts into scalable and clinically relevant drug delivery platforms.

## Figures and Tables

**Table 4:** Analytical techniques for characterization of terpenoid phytosomes.

Parameter	Analytical Technique	Key Observations	References
Particle Size and Distribution	Dynamic Light Scattering (DLS)	Ideal PDI < 0.3 for stable systems	[62]
Surface Charge	Electrophoretic Mobility (Zeta Potential)	High absolute values (>±30 mV) indicate good stability	[63]
Surface Morphology	TEM	Direct visual confirmation of the spherical shape	[65]
Chemical Interaction	ATR-FTIR Spectroscopy	Shift or disappearance of –OH or C=O stretching vibrations, indicating hydrogen bonding	[61]
Thermodynamic State	DSC (Differential Scanning Calorimetry)	Loss of the drug’s endothermic melting peak, confirming its molecular dispersion in the lipid matrix	[66]
Crystallinity	Powder XRD (X-ray Diffraction)	Transition from sharp crystalline peaks to a broad “halo” pattern (amorphous form)	[64]
Complexation Efficiency	HPLC	Measurement of the Drug Entrapment Efficiency (EE%) and Loading Capacity (LC%)	[67]
Molecular Environment	^1^H-NMR	Analysis of chemical shifts to pinpoint specific atomic interactions between terpenoid and PC	[68]

**Table 6:** Current stages of terpenoid phytosomes research.

Therapeutic Area	Model	Key Outcome	Reference
Inflammation	Clinical trial	↓ TNF-α, 5-Lipoxygenase	[44,45,48,50,56,57,75]
Cancer	In vitro, in vivo	↑ apoptosis	[51]
Wound healing	In vivo	Accelerated closure	[53]
Gastrointestinal disorders (ulcerative colitis, irritable bowel syndrome)	Clinical trial	Pain relief	[47]
Muscoskeletal disorders (knee pain)	Clinical trial	↓ inflammatory biomarkers (CRP) and ↓ cartilage damage markers (COMP)	[50]
Asthma	Clinical trial	Reduces the need for inhalation therapy ICS + LABA	[57]

## Data Availability

No new data were created or analyzed in this study. Data sharing is not applicable to this article.

## References

[1] Brahmkshatriya P.P., Brahmkshatriya P.S. (2013). Terpenes: Chemistry, Biological Role, and Therapeutic Applications. Natural Products Phytochemistry, Botany and Metabolism of Alkaloids, Phenolics and Terpenes.

[2] Pouton C.W., Porter C.J.H. (2008). Formulation of Lipid-Based Delivery Systems for Oral Administration: Materials, Methods and Strategies. Adv. Drug Deliv. Rev..

[3] Bombardelli E., Curri S.B., Della Loggia R., Del Negro P., Tubaro A., Gariboldi P. (1989). Complexes between Phospholipids and Vegetal Derivatives of Biological Interest. Fitoterapia.

[4] Benedini L.A., Messina P.V. (2025). Smart Vesicle Therapeutics: Engineering Precision at the Nanoscale. Pharmaceutics.

[5] Tikhonova E.V., Adekenov S.M., Samenov N.A., Gil’manov M.K. (2001). Production of Lipoglycoprotein Micelles with Arglabine Antitumor Preparation. Chem. Nat. Compd..

[6] Tikhonova E.V., Adekenov S.M., Samenov N.A., Gil’manov M.K. Method for Producing a Liposomal Anticancer Agent. Kazakhstan Preliminary Patent 10870, Application No. 990677.1, filed 10 June 1999, published 15 November 2001. https://gosreestr.kazpatent.kz/Invention/Details?docNumber=132280.

[7] Gabizon A.A., Gabizon-Peretz S., Modaresahmadi S., La-Beck N.M. (2025). Thirty Years from FDA Approval of Pegylated Liposomal Doxorubicin (Doxil/Caelyx): An Updated Analysis and Future Perspective. BMJ Oncol..

[8] Nematollahi M.H., Pardakhty A., Torkzadeh-Mahanai M., Mehrabani M., Asadikaram G. (2017). Changes in Physical and Chemical Properties of Niosome Membrane Induced by Cholesterol: A Promising Approach for Niosome Bilayer Intervention. RSC Adv..

[9] Bautista-Solano A.A., Dávila-Ortiz G., Perea-Flores M.d.J., Martínez-Ayala A.L. (2025). A Comprehensive Review of Niosomes: Composition, Structure, Formation, Characterization, and Applications in Bioactive Molecule Delivery Systems. Molecules.

[10] Aparajay P., Dev A. (2022). Functionalized Niosomes as a Smart Delivery Device in Cancer and Fungal Infection. Eur. J. Pharm. Sci..

[11] Cevc G., Blume G. (1992). Lipid Vesicles Penetrate into Intact Skin Owing to the Transdermal Osmotic Gradients and Hydration Force. Biochim. Biophys. Acta-Biomembr..

[12] Touitou E., Dayan N., Bergelson L., Godin B., Eliaz M. (2000). Ethosomes—Novel Vesicular Carriers for Enhanced Delivery: Characterization and Skin Penetration Properties. J. Control. Release.

[13] Spicer P. (2005). Cubosome Processing: Industrial Nanoparticle Technology Development. Chem. Eng. Res. Des..

[14] Pattni B.S., Chupin V.V., Torchilin V.P. (2015). New Developments in Liposomal Drug Delivery. Chem. Rev..

[15] Sharmila A., Bhadra P., Kishore C., Selvaraj C.I., Kavalakatt J., Bishayee A. (2025). Nanoformulated Terpenoids in Cancer: A Review of Therapeutic Applications, Mechanisms, and Challenges. Cancers.

[16] Toma L., Deleanu M., Sanda G.M., Barbălată T., Niculescu L.Ş., Sima A.V., Stancu C.S. (2024). Bioactive Compounds Formulated in Phytosomes Administered as Complementary Therapy for Metabolic Disorders. Int. J. Mol. Sci..

[17] Ortega-Pérez L.G., Ayala-Ruiz L.A., Magaña-Rodríguez O.R., Piñón-Simental J.S., Aguilera-Méndez A., Godínez-Hernández D., Rios-Chavez P. (2023). Development and Evaluation of Phytosomes Containing Callistemon Citrinus Leaf Extract: A Preclinical Approach for the Treatment of Obesity in a Rodent Model. Pharmaceutics.

[18] Hüsch J., Bohnet J., Fricker G., Skarke C., Artaria C., Appendino G., Schubert-Zsilavecz M., Abdel-Tawab M. (2013). Enhanced Absorption of Boswellic Acids by a Lecithin Delivery Form (Phytosome^®^) of Boswellia Extract. Fitoterapia.

[19] Wu S., Lu J. (2025). Liposome-Enabled Nanomaterials for Muscle Regeneration. Small Methods.

[20] Bartelds R., Nematollahi M.H., Pols T., Stuart M.C.A., Pardakhty A., Asadikaram G., Poolman B. (2018). Niosomes, an Alternative for Liposomal Delivery. PLoS ONE.

[21] Huang Y., Xie F.J., Cao X., Li M.Y. (2021). Research Progress in Biosynthesis and Regulation of Plant Terpenoids. Biotechnol. Biotechnol. Equip..

[22] Atriya A., Majee C., Mazumder R., Choudhary A.N., Salahuddin, Mazumder A., Dahiya A., Priya N. (2023). Insight into the Various Approaches for the Enhancement of Bioavailability and Pharmacological Potency of Terpenoids: A Review. Curr. Pharm. Biotechnol..

[23] Guo J., Huang M., Hou S., Yuan J., Chang X., Gao S., Zhang Z., Wu Z., Li J. (2024). Therapeutic Potential of Terpenoids in Cancer Treatment: Targeting Mitochondrial Pathways. Cancer Rep..

[24] Barani M., Sangiovanni E., Angarano M., Rajizadeh M.A., Mehrabani M., Piazza S., Gangadharappa H.V., Pardakhty A., Mehrbani M., Dell’agli M. (2021). Phytosomes as Innovative Delivery Systems for Phytochemicals: A Comprehensive Review of Literature. Int. J. Nanomed..

[25] Albash R., Al-Mahallawi A.M., Hassan M., Alaa-Eldin A.A. (2021). Development and Optimization of Terpene-Enriched Vesicles (Terpesomes) for Effective Ocular Delivery of Fenticonazole Nitrate: In Vitro Characterization and in Vivo Assessment. Int. J. Nanomed..

[26] Wu H., Wang B., Li X., Lu C., Zeng Q., Lu L., Chen M., Wu Y. (2024). Development of Ampelopsis Radix Ethanolic Extract Loaded Phytosomes for Improved Efficacy in Colorectal Cancer: In Vitro and In Vivo Assessment Study. J. Oleo Sci..

[27] Mazumder A., Dwivedi A., Du Preez J.L., Du Plessis J. (2016). In Vitro Wound Healing and Cytotoxic Effects of Sinigrin-Phytosome Complex. Int. J. Pharm..

[28] Lu M., Qiu Q., Luo X., Liu X., Sun J., Wang C., Lin X., Deng Y., Song Y. (2019). Phyto-Phospholipid Complexes (Phytosomes): A Novel Strategy to Improve the Bioavailability of Active Constituents. Asian J. Pharm. Sci..

[29] Roy S., Ghosh A., Majie A., Karmakar V., Das S., Dinda S.C., Bose A., Gorain B. (2024). Terpenoids as Potential Phytoconstituent in the Treatment of Diabetes: From Preclinical to Clinical Advancement. Phytomedicine.

[30] Neamatallah T., Malebari A.M., Alamoudi A.J., Nazreen S., Alam M.M., Bin-Melaih H.H., Abuzinadah O.A., Badr-Eldin S.M., Alhassani G., Makki L. (2023). Andrographolide Nanophytosomes Exhibit Enhanced Cellular Delivery and Pro-Apoptotic Activities in HepG2 Liver Cancer Cells. Drug Deliv..

[31] Gorter K. (1911). Le Principe Amer de l’Andrographis Paniculata Nees. Recl. Trav. Chim. Pays-Bas Belg..

[32] Rajani M., Shrivastava N., Ravishankara M.N. (2000). A Rapid Method for Isolation of Andrographolide from Andrographis Paniculata Nees (Kalmegh). Pharm. Biol..

[33] Adekenov S.M. (2016). Chemical Modification of Arglabin and Biological Activity of Its New Derivatives. Fitoterapia.

[34] Subbaraju G.V., Sridhar P., Ramakrishna S., Sreemannarayana A., Vanisree M., Kanna Babu S. (2004). Isolation and HPLC Estimation of Six Boswellic Acids from Boswellia Serrata Extract. Asian J. Chem..

[35] Culioli G., Mathe C., Archier P., Vieillescazes C. (2003). A Lupane Triterpene from Frankincense (Boswellia Sp., Burseraceae). Phytochemistry.

[36] Beton J.L., Halsall T.G., Jones E.R.H. (1956). The Chemistry of Triterpenes and Related Compounds. Part Xxviii.* β-Boswellic Acid. J. Chem. Soc..

[37] Pardhy R.S., Bhattacharyya S.C. (1978). Beta-Boswellic Acid, Acetyl-Beta-Boswellic Acid, Acetyl-11-Keto-Beta-Boswellic Acid & 11-Keto-Beta-Boswellic Acid, Four Pentacyclic Triterpene Acids from the Resin of Boswellia Serrata Roxb. Indian J. Chem. Sect. B Org. Chem. Incl. Med. Chem..

[38] Iram F., Khan S.A., Husain A. (2017). Phytochemistry and Potential Therapeutic Actions of Boswellic Acids: A Mini-Review. Asian Pac. J. Trop. Biomed..

[39] Kekulé A. (1872). Ueber Die Constitution Des Carvacrols. Ber. Dtsch. Chem. Ges..

[40] Suntres Z.E., Coccimiglio J., Alipour M. (2015). The Bioactivity and Toxicological Actions of Carvacrol. Crit. Rev. Food Sci. Nutr..

[41] J.S.H. (1932). Wax-like Coating of the Apple. J. Frankl. Inst..

[42] Woźniak Ł., Skąpska S., Marszałek K. (2015). Ursolic Acid—A Pentacyclic Triterpenoid with a Wide Spectrum of Pharmacological Activities. Molecules.

[43] Riva A., Allegrini P., Franceschi F., Togni S., Giacomelli L., Eggenhoffner R. (2017). Casperome^®^ in the Management of Musculoskeletal Disorders: A Review. Eur. Rev. Med. Pharmacol. Sci..

[44] Pellegrini L., Milano E., Franceschi F., Belcaro G., Gizzi G., Feragalli B., Dugall M., Luzzi R., Togni S., Eggenhoffner R. (2016). Managing Ulcerative Colitis in Remission Phase: Usefulness of Casperome^®^, an Innovative Lecithin-Based Delivery System of Boswellia Serrata Extract. Eur. Rev. Med. Pharmacol. Sci..

[45] Sharma A., Gupta N.K., Dixit V.K. (2010). Complexation with Phosphatidyl Choline as a Strategy for Absorption Enhancement of Boswellic Acid. Drug Deliv..

[46] Gomaa A.A., Mohamed H.S., Abd-ellatief R.B., Gomaa M.A. (2021). Boswellic Acids/Boswellia Serrata Extract as a Potential COVID-19 Therapeutic Agent in the Elderly. Inflammopharmacology.

[47] Belcaro G., Gizzi G., Pellegrini L., Corsi M., Dugall M., Cacchio M., Feragalli B., Togni S., Riva A., Eggenhoffner R. (2017). Supplementation with a Lecithin-Based Delivery Form of Boswellia Serrata Extract (Casperome^®^) Controls Symptoms of Mild Irritable Bowel Syndrome. Eur. Rev. Med. Pharmacol. Sci..

[48] Sgaramella L.I., Pasculli A., Moschetta M., Puntillo F., Dicillo P., Clemente L., Maruccia M., Mastropasqua M.G., Piombino M., Resta N. (2025). Randomized Controlled Trial of Bromelain and Alpha-Lipoic Acid in Breast Conservative Surgery. Sci. Rep..

[49] Di Giacomo P., Forte G., Capogna I., Casagrande M., Di Paolo C. (2024). The Role of Nutraceuticals in the Management of Temporomandibular Disorders. J. Complement. Integr. Med..

[50] Franceschi F., Togni S., Belcaro G., Dugall M., Luzzi R. (2016). A Novel Lecithin Based Delivery Form of Boswellic Acids (Casperome^®^) for the Management of Osteo-Muscular Pain: A Registry Study in Young Rugby Players. Eur. Rev. Med. Pharmacol. Sci..

[51] Poudel K., Gautam M., Maharjan S., Jeong J., Choi H. (2020). Dual Stimuli-Responsive Ursolic Acid-Embedded Nanophytoliposome for Targeted Antitumor Therapy. Int. J. Pharm..

[52] Biswas S., Mukherjee P.K., Harwansh R.K., Bannerjee S., Bhattacharjee P. (2019). Enhanced Bioavailability and Hepatoprotectivity of Optimized Ursolic Acid–Phospholipid Complex. Drug Dev. Ind. Pharm..

[53] Tafish A.M., El-Sherbiny M., Al-Karmalawy A.A., Soliman O.A.E.A., Saleh N.M. (2023). Carvacrol-Loaded Phytosomes for Enhanced Wound Healing: Molecular Docking, Formulation, DoE-Aided Optimization, and in Vitro/in Vivo Evaluation. Int. J. Nanomed..

[54] Khalil W.A., Elkhamy S.A., Hegazy M.M., Hassan M.A.E., Tafish A.M., Abdelnour S.A., El-Harairy M.A. (2025). Beneficial Effects of Carvacrol Loaded Phytosomes on Enhancing Cryotolerance of Buffalo Semen Following Cryopreservation. Sci. Rep..

[55] Ren Z., Chen Z., Xie Y., Coghi P. (2025). Andrographolide and Its Derivatives: A Comprehensive Review of Anti-Infective Properties and Clinical Potential. Molecules.

[56] Giacosa A., Riva A., Petrangolini G., Allegrini P., Fazia T., Bernardinelli L., Peroni G., Rondanelli M. (2022). Positive Effects of a Lecithin-Based Delivery Form of Boswellia Serrata Extract in Acute Diarrhea of Adult Subjects. Nutrients.

[57] Ferrara T., De Vincentiis G., Di Pierro F. (2015). Functional Study on Boswellia Phytosome as Complementary Intervention in Asthmatic Patients. Eur. Rev. Med. Pharmacol. Sci..

[58] Kapse M.V., Mulla J.A.S. (2024). Unlocking the Potential of Phytosomes: A Review of Formulation Techniques, Evaluation Methods, and Emerging Applications. Acta Mater. Medica.

[59] Talebi M., Shahbazi K., Dakkali M.S., Akbari M., Almasi Ghale R., Hashemi S., Sashourpour M., Mojab F., Aminzadeh S. (2025). Phytosomes: A Promising Nanocarrier System for Enhanced Bioavailability and Therapeutic Efficacy of Herbal Products. Phytomed. Plus.

[60] Pilato S., Carradori S., Melfi F., Di Giacomo S., Ciavarella S., Ciulla M., Fontana A., Di Profio P., Aschi M., Moffa S. (2025). Phenolic Terpenes in Liposomal Bilayers: Unraveling Physicochemical Interactions and Membrane Perturbation via Biophysical and Computational Approaches. J. Colloid Interface Sci..

[61] Socaciu C., Fetea F., Socaciu M.A. (2024). Synthesis and Characterization of PEGylated Liposomes and Nanostructured Lipid Carriers with Entrapped Bioactive Triterpenoids: Comparative Fingerprints and Quantification by UHPLC-QTOF-ESI(+)-MS, ATR-FTIR Spectroscopy, and HPLC-DAD. Pharmaceuticals.

[62] Ragheb R., Nobbmann U. (2020). Multiple Scattering Effects on Intercept, Size, Polydispersity Index, and Intensity for Parallel (VV) and Perpendicular (VH) Polarization Detection in Photon Correlation Spectroscopy. Sci. Rep..

[63] Clogston J.D., Patri A.K. (2011). Zeta Potential Measurement. Methods Mol. Biol..

[64] Elendran S., Shiva Kumar V., Sundralingam U., Tow W.-K., Palanisamy U.D. (2024). Enhancing the Bioavailability of the Ellagitannin, Geraniin: Formulation, Characterization, and in Vivo Evaluation. Int. J. Pharm..

[65] Ďúranová H., Kšiňan S., Kuželová L., Šimora V., Ďurišová Ľ., Olexíková L., Ernst D., Kolenčík M. (2024). Nanoparticle-Plant Interactions: Physico-Chemical Characteristics, Application Strategies, and Transmission Electron Microscopy-Based Ultrastructural Insights, with a Focus on Stereological Research. Chemosphere.

[66] Zambrano P., Manrique-Moreno M., Petit K., Colina J.R., Jemiola-Rzeminska M., Suwalsky M., Strzalka K. (2024). Differential Scanning Calorimetry in Drug-Membrane Interactions. Biochem. Biophys. Res. Commun..

[67] Tsarenko E., Schubert U.S., Nischang I. (2023). Nanoparticle Formulation Composition Analysis by Liquid Chromatography on Reversed-Phase Monolithic Silica. Anal. Chem..

[68] Vu H.T.H., Hook S.M., Siqueira S.D., Müllertz A., Rades T., McDowell A. (2018). Are Phytosomes a Superior Nanodelivery System for the Antioxidant Rutin?. Int. J. Pharm..

[69] Semalty A., Semalty M., Rawat M.S.M., Franceschi F. (2010). Supramolecular Phospholipids-Polyphenolics Interactions: The PHYTOSOME^®^ Strategy to Improve the Bioavailability of Phytochemicals. Fitoterapia.

[70] Deleanu M., Toma L., Sanda G.M., Barbălată T., Niculescu L.Ş., Sima A.V., Deleanu C., Săcărescu L., Suciu A., Alexandru G. (2023). Formulation of Phytosomes with Extracts of Ginger Rhizomes and Rosehips with Improved Bioavailability, Antioxidant and Anti-Inflammatory Effects In Vivo. Pharmaceutics.

[71] Xu F., Xu S., Yang L., Qu A., Li D., Yu M., Wu Y., Zheng S., Ruan X., Wang Q. (2024). Preparing a Phytosome for Promoting Delivery Efficiency and Biological Activities of Methyl Jasmonate-Treated Dendropanax Morbifera Adventitious Root Extract (DMARE). Biomolecules.

[72] Dewi M.K., Muhaimin M., Joni I.M., Hermanto F., Chaerunisaa A.Y. (2024). Fabrication of Phytosome with Enhanced Activity of Sonneratia Alba: Formulation Modeling and in Vivo Antimalarial Study. Int. J. Nanomed..

[73] Islam N., Irfan M., Hussain T., Mushtaq M., Khan I.U., Yousaf A.M., Ghori M.U., Shahzad Y. (2022). Piperine Phytosomes for Bioavailability Enhancement of Domperidone. J. Liposome Res..

[74] El-Menshawe S.F., Ali A.A., Rabeh M.A., Khalil N.M. (2018). Nanosized Soy Phytosome-Based Thermogel as Topical Anti-Obesity Formulation: An Approach for Acceptable Level of Evidence of an Effective Novel Herbal Weight Loss Product. Int. J. Nanomed..

[75] Hüsch J., Gerbeth K., Fricker G., Setzer C., Zirkel J., Rebmann H., Schubert-Zsilavecz M., Abdel-Tawab M. (2012). Effect of Phospholipid-Based Formulations of Boswellia Serrata Extract on the Solubility, Permeability, and Absorption of the Individual Boswellic Acid Constituents Present. J. Nat. Prod..

[76] Abdihaji M., Chegeni M.M., Hadizadeh A., Farrokhzad N., Kheradmand Z., Fakhrfatemi P., Faress F., Moeinabadi-Bidgoli K., Noorbazargan H., Mostafavi E. (2023). Polyvinyl Alcohol (PVA)-Based Nanoniosome for Enhanced in Vitro Delivery and Anticancer Activity of Thymol. Int. J. Nanomed..

[77] Dutt Y., Pandey R.P., Dutt M., Gupta A., Vibhuti A., Raj V.S., Chang C.M., Priyadarshini A. (2023). Liposomes and Phytosomes: Nanocarrier Systems and Their Applications for the Delivery of Phytoconstituents. Coord. Chem. Rev..

[78] Mehmood H. (2023). Phytosome as a Novel Carrier for Delivery of Phytochemicals: A Comprehensive Review. Middle East J. Appl. Sci. Technol..

[79] Kaps A., Gwiazdoń P., Chodurek E. (2021). Nanoformulations for Delivery of Pentacyclic Triterpenoids in Anticancer Therapies. Molecules.

